# Glutamine Administration Attenuates Kidney Inflammation in Obese Mice Complicated with Polymicrobial Sepsis

**DOI:** 10.1155/2021/5597118

**Published:** 2021-03-30

**Authors:** Li-Han Su, Ming-Tsan Lin, Sung-Ling Yeh, Chiu-Li Yeh

**Affiliations:** ^1^School of Nutrition and Health Sciences, College of Nutrition, Taipei Medical University, Taipei, Taiwan; ^2^Department of Surgery, National Taiwan University Hospital and College of Medicine, National Taiwan University, Taipei, Taiwan

## Abstract

Obesity is a well-known public health issue around the world. Sepsis is a lethal clinical syndrome that causes multiorgan failure. Obesity may aggravate inflammation in septic patients. Glutamine (GLN) is a nutrient with immune regulatory and anti-inflammatory properties. Since sepsis is a common contributing factor for acute kidney injury (AKI), this study investigated the effects of GLN administration on sepsis-induced inflammation and AKI in obese mice. A high-fat diet which consists of 60% of calories from fat was provided for 10 weeks to induce obesity in the mice. Then, the obese mice were subdivided into sepsis with saline (SS) or GLN (SG) groups. Cecal ligation and puncture (CLP) was performed to produce sepsis. The SS group was intravenously injected with saline while the SG group was administered GLN one or two doses after CLP. Obese mice with sepsis were sacrificed at 12, 24, or 48 h post-CLP. Results revealed that sepsis resulted in upregulated high-mobility group box protein-1 pathway-associated gene expression in obese mice. Also, expressions of macrophage/neutrophil infiltration markers and inflammatory cytokines in kidneys were elevated. Obese mice treated with GLN after sepsis reversed the depletion of plasma GLN, reduced production of lipid peroxides, and downregulated macrophage/neutrophil infiltration and the inflammatory-associated pathway whereas tight junction gene expression increased in the kidneys. These findings suggest that intravenously administered GLN to obese mice after sepsis alleviated inflammation and attenuated AKI. This model may have clinical application to obese patients with a risk for infection in abdominal surgery.

## 1. Introduction

Obesity is an important public health issue worldwide because of its high global prevalence. Obesity is mainly caused by high calorie intake and deficiency of physical activity that develop gradually with cellular physiologic changes before the symptoms of diseases become apparent. Obese individuals present systemic markers of chronic low-grade inflammation. Also, obesity results in dysregulation of immune responses [1]. These changes are closely associated with metabolic disturbances that may have adverse impact on host immunity during infection and promote disease progression [1, 2]. Obese subjects are considered frail with increased risk of recurrent nosocomial infections [2]. In the critically ill population, a previous study found that compared to normal weight, obese patients have higher risk of infectious complications that lead to sepsis [3].

Sepsis is a lethal clinical syndrome with multiorgan failure [4]. It is considered that the dysregulated immune reaction in response to infection results in dysregulation between pro- and anti-inflammatory responses that ultimately lead to irreversible multiorgan failure [5]. For critically ill patients, acute kidney injury (AKI) is an independent risk factor for mortality, and the most common contributing factor for AKI is sepsis [6]. The pathogenesis of sepsis-induced AKI is complicated and multifactorial. Although both the pro- and anti-inflammatory mechanisms are involved, inflammation is the key component and proinflammatory cytokine production can be used as predictor of AKI among septic patients [7]. Previous studies found that there were different reactions between obese and lean animals in respect to the septic insult. Obese animals exhibited more severe symptoms than the lean ones and suggest that compared with the lean animals, obesity exaggerates inflammatory response during sepsis [8–10]. Obesity was considered to be an independent risk factor for mortality in critically ill populations [11] and has become a major concern in septic patients.

Glutamine (GLN) is a nutrient with anti-inflammatory and immunomodulatory properties. Although a multicenter clinical study revealed that GLN administration was associated with an increased mortality among critical patients with multiorgan failure [12], subsequent clinical trials found that parenteral GLN supplementation is safe in surgical intensive care unit (ICU) patients and improved outcomes in stabilized patients with organ function [13, 14]. Previous studies performed by our laboratory revealed that GLN administration attenuates inflammatory reaction and remote organ injury in sepsis [15–17]. GLN was found to elicit a more-balanced T helper cell polarization [16], decreased programmed cell death 1 expression on immune cells [18], and downregulated high-mobility group box protein-1- (HMGB-1-) mediated pathway [19], thus alleviating kidney injury in sepsis. However, the metabolic stress derived from obesity exposed to an acute inflammatory stimulus, such as sepsis, is different from sepsis alone. As we know, there is no study investigating the impact of GLN on inflammation and AKI in obesity complicated with sepsis. This study used a high-fat diet to induce obesity; thereafter, cecal ligation and puncture (CLP) was performed to create a mouse model of obesity with polymicrobial sepsis. We hypothesized that treatment with GLN after sepsis may have beneficial effects on attenuating inflammatory response and subsequent AKI in obese mice concurrent with sepsis.

## 2. Materials and Methods

### 2.1. Animals

Male C57BL/6 mice (5 weeks old with ~20 g in body weight) were used in this study. Mice were housed in the Laboratory Animal Center at the Taipei Medical University (TMU; Taipei, Taiwan). The animal center conditions were21 ± 2°Cand relative humidity of 50% ~55% with a 12 h light-dark cycle. Standard rodent chow diet (Purina no. 5001, Fort Worth, TX, USA) and water were provided *ad libitum* during the acclimation period. Care and use of laboratory animals were in compliance with the *Guide for the Care and Use of Laboratory Animals* [20]. The experimental protocols were approved by the Animal Care and Use Committee of TMU.

### 2.2. Experimental Procedures

In the beginning, mice were randomly assigned to a normal control (NC, *n* = 6) group and a high-fat (HF, *n* = 60) group. Mice in the NC group were provided standard rodent chow diet, while those in the HF group were fed a high-fat diet which consists of 60% kcal as fat [21] for 10 weeks. The composition of the diet is supplied by a commercial company (Research Diets, New Brunswick, NJ, USA) as shown in Table 1. After 10 weeks of feeding, mice in the HF group were subdivided into sepsis with saline (SS, *n* = 30) and sepsis with GLN (SG, *n* = 30) groups, then subjected to CLP as previously described [22]. Mice were injected intraperitoneally with Zoletil (25 mg/kg body weight (BW), Virbac, Carros, France) and Rompun (10 mg/kg BW, Bayer, Leverkusen, Germany) for anesthetization. To open the peritoneum, the abdominal wall was incised with about a 1 cm incision. Then, the cecum was ligated at 50% below the ileocecal valve and was punctured through with a 23-gauge needle. A small drop of feces was squeezed out and smeared onto the abdomen. The abdomen was closed with continuous suture. The CLP operation in all animals was performed by the same technician to ensure consistency. After the operation, sterile saline (4 mL/kg BW) was subcutaneously rehydrated and free access to water and rodent chow. One hundred microliters of 0.25% bupivacaine was administered at the incision site before skin closure to relieve pain after the surgery. Mice in the SS and SG groups were sacrificed at 12, 24, or 48 h post-CLP, respectively, according to their schedule. Mice sacrificed at 12 and 24 h after CLP were injected with a single dose of either saline or GLN (0.75 g GLN/kg BW) intravenously via a tail vein 1 h after CLP. GLN was provided as alanyl-glutamine dipeptide (Dipeptiven; Fresenius Kabi, Homburg, Germany). This dosage was previously shown to have immunomodulatory effects on sepsis [18, 23]. Mice euthanized at 48 h were injected with another dose of saline or GLN 24 h after the first injection to enhance the efficacy of GLN. At the end of the experiment, mice were anesthetized and euthanized by cardiac puncture. Body weight and epididymal tissue weights were recorded. Blood samples were collected and centrifuged to obtain plasma. The peritoneum was opened and irrigated with saline to obtain peritoneal lavage fluid (PLF). Kidneys were excised. All the samples were stored at −80°C for further analysis.

### 2.3. Measurements of Biochemical Markers and Chemokines in Plasma

Kidney function markers (blood urea nitrogen (BUN) and creatinine (Cre)) were analyzed by the VetTest® Chemistry Analyzer (IDEXX Laboratories Inc., Westbrook, MN, USA). Kidney injury markers (neutrophil gelatinase-associated lipocalin (NGAL)) were measured using a commercially available enzyme-linked immunosorbent assay (ELISA) kit (R&D Systems Inc., Minneapolis, MN, USA). Inflammatory chemokines (monocyte chemoattractant protein-1 (MCP-1) and keratinocyte-derived chemokine (KC)) were also measured by ELISA kits (R&D Systems Inc., Minneapolis, MN, USA). Antibodies (Abs) specific to mouse NGAL, MCP-1, and KC were precoated on the wells of microtiter strips. Plasma samples were incubated and developed with reagents. The absorbance of each well was measured by a spectrophotometer. The analyzing procedures were instructed by the protocols provided by the manufacturer.

### 2.4. Measurement of Plasma Amino Acid Concentrations

A Waters AccQ-Tag derivatization kit (Milford, MA, USA) was used to prepare the plasma samples. Using the ACQUITY UPLC System (Waters), the samples were applied to ultraperformance liquid chromatography (UPLC) for separation. A Xevo TQ-XS (Waters) mass spectrometer was used for monitoring. The amino acid concentrations were measured by Waters MassLynx 4.2 software and quantified by TargetLynx.

### 2.5. Inflammatory Mediator Concentrations in PLF

Interleukin-10 (IL-10), tumor necrosis factor-*α* (TNF-*α*), MCP-1, and KC were measured by ELISA kits in a microtiter plate (R&D Systems Inc., Minneapolis, MN, USA). Details are mentioned above.

### 2.6. Messenger (m)RNA Extraction and Analysis of a Real-Time Reverse-Transcription (RT) Quantitative Polymerase Chain Reaction (qPCR)

Kidney tissues were homogenized and total RNA was isolated by a TRIzol reagent (Invitrogen, Carlsbad, CA, USA). RNA pellets were dissolved in RNase-free water and stored at -80°C for further analysis. Using a spectrophotometer, RNA concentrations were quantified at absorbances of 260 and 280 nm. We used a RevertAid™ first-strand complementary (c)DNA synthesis kit (Thermo Fisher Scientific, Vilnius, Lithuania) to synthesize cDNA. cDNA was stored at -80°C until being used. RT was performed by subsequent incubation for 5 min at 65°C, 60 min at 42°C, and 5 min at 70°C. Messenger RNA genes were amplified by a real-time RT-PCR using the 7300 Real-Time PCR System (Applied Biosystems, Foster City, CA, USA) with SYBR Green I as the detection format. Genes measured in kidney tissues included the high-mobility group box protein (HMGB)-1-associated pathway (HMGB-1, myeloid differentiation factor (MyD)88, toll-like receptor- (TLR-) 4, and nuclear factor- (NF-) *κ*B), inflammatory cytokines and chemokines (IL-6, TNF-*α*, and MCP-1), macrophage infiltration markers (cluster of differentiation (CD)68 and epidermal growth factor-like module-containing mucin-like hormone receptor-like-1 (EMR-1)), kidney injury molecule-1 (Kim-1), and tight junction protein zonula occluden-1 (ZO-1). Primers used are listed in Table 2. All primers were provided by Mission Biotech (Taipei, Taiwan) based on deposited cDNA sequences (GenBank database, NCBI). A total volume of 25 *μ*L containing Maxima SYBR Green/ROX qPCR Master Mix (2x) (Thermo Fisher Scientific), 100 ng of cDNA, and 40 nM of each primer was used for amplification. The reaction was processed by one cycle of 2 min at 50°C and 10 min at 95°C, followed by 40 cycles of 15 s at 95°C and 1 min at 60°C, and a final dissociation curve (DC) was analyzed. The gene expression levels were quantified in duplicate by means of a real-time RT-PCR. The relative mRNA expression was calculated by cycle threshold (CT) values and normalized to mouse glyceraldehyde 3-phosphate dehydrogenase (GAPDH).

### 2.7. Analysis of Myeloperoxidase (MPO) Activity in Kidney Homogenates

Kidney tissue were washed with cold phosphate-buffered saline (PBS) and then homogenized with MPO assay buffer (1 : 10, wt/vol). The homogenates were centrifuged for 10 min at 13,000 g to remove insoluble materials. The supernatants were collected and measured by Myeloperoxidase Activity Assay kit (Abcam, Cambridge, MA, USA). The reagents and samples were added to the 96-well plate and incubated for 1 hr. MPO activities were determined at 412 nm optical density using a spectrophotometer and expressed as a unit/mg protein [23]. The analyzing procedures were instructed by the protocols provided by the manufacturer. A Bradford protein assay reagent kit (Bio-Rad, Richmond, CA) was used to measure protein concentrations.

### 2.8. Analysis of Thiobarbituric Acid Reactive Substance (TBARS) in Kidney Tissues

Kidney tissues were homogenized at 4°C in a reagent prepared by protease and phosphatase inhibitor (Thermo Fisher Scientific, Vilnius, Lithuania) and Tissue Protein Extraction Reagent (T-PER™, Thermo Fisher Scientific) (1 : 100). The homogenates were centrifuged at 12,000 rpm for 10 min. The supernatants were used for quantifying TBARS. The TBARS consists of malondialdehyde (MDA) and thiobarbituric acid-reacted lipid peroxidation end products. TBARS levels were analyzed by a commercial assay kit (Cayman, MI, USA) and determined at 530-540 nm optical density. The concentrations of TBARS were expressed as *μ*M/mg protein. The Bradford protein assay kit (Bio-Rad) was used to analyze protein concentrations.

### 2.9. Statistical Analysis

All data are presented as the mean ± standard error of the mean (SEM). Data were analyzed with the GraphPad Prism 5 statistical software program (GraphPad Software, La Jolla, CA, USA). Differences between 2 sepsis groups at the same time point were analyzed by *t*-test. The comparison among NC and the sepsis groups at three different time points was analyzed by a one-way analysis of variance (ANOVA) followed by Tukey's post hoc test. A *p* value of <0.05 was considered statistically significant.

## 3. Results

### 3.1. Changes in Epididymal Adipose Tissue and Body Weights after High-Fat Diet Feeding

The initial BWs did not differ between the NC and HF groups. After 10 weeks of feeding, mice in the HF group had higher epididymal fat weights (NC 0.63 ± 0.02 g vs. HF 2.54 ± 0.09 g, *p* < 0.0001) than those in the NC group. Also, BWs were higher in the HF group than in the NC group (NC 26.4 ± 0.7 g vs. HF 36.5 ± 1.1 g, *p* < 0.0001).

### 3.2. Plasma Concentrations of Biochemical Markers and Inflammatory Chemokines

Plasma BUN, Cre, NGAL, KC, and MCP-1 concentrations were significantly elevated after CLP. There were no differences in BUN, Cre, and MCP-1 between the SS and SG groups at 12, 24, or 48 h post-CLP. NGAL levels in the SG group were significantly lower at 24 h and KC were lower at 12 h than those in the SS group post-CLP (Table 3).

### 3.3. Plasma Amino Acid Concentrations

**Figure 1:**
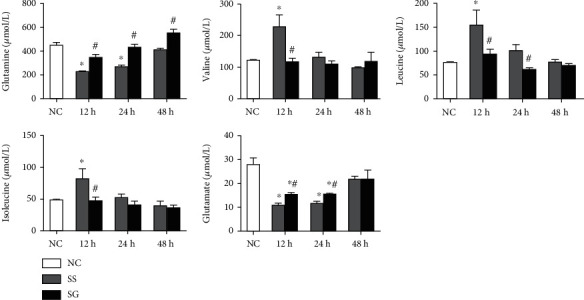
Plasma amino acid concentrations in the normal control (NC) and the sepsis groups at different time points. SS: sepsis group with saline; SG: sepsis group with glutamine. Values are expressed as the mean ± SEM. All data are representative of duplicate measurements at 12, 24, and 48 h after cecal ligation and puncture (CLP) (*n* = 8 for each respective group). Differences between 2 sepsis groups at the same time point were analyzed by *t*-test. The comparison among NC and the sepsis groups at three different time points were analyzed by a one-way analysis of variance (ANOVA) followed by Tukey's post hoc test. ^∗^Significantly differs from the NC group; ^#^significantly differs from the SS group at the same time point (*p* < 0.05).

Glutamate, GLN, and branch-chain amino acids (BCAAs) including leucine, valine, and isoleucine levels were measured at 12, 24, and 48 h after CLP. The SS group had lower glutamate and GLN concentrations at 12 and 24 h whereas the BCAAs were higher at 12 h post-CLP when compared to those of the NC group. The SG group exhibited higher glutamate levels at 12 and 24 h and GLN at all the three time points than the SS group after CLP. The isoleucine and valine concentrations were lower at 12 h, while leucine at 12 and 24 h after CLP in the SG group than those in the SS groups (Figure 1).

### 3.4. PLF Cytokine and Chemokine Levels

Sepsis resulted in elevation of TNF-*α*, IL-10, KC, and MCP-1 concentrations in obese mice. Compared to the SS group, the SG group exhibited lower TNF-*α* at 48 h and KC and MCP-1 at 12 h whereas anti-inflammatory IL-10 was elevated at 24 h after CLP (Table 4).

### 3.5. Inflammatory Gene Expressions in the Kidney

**Figure 2:**
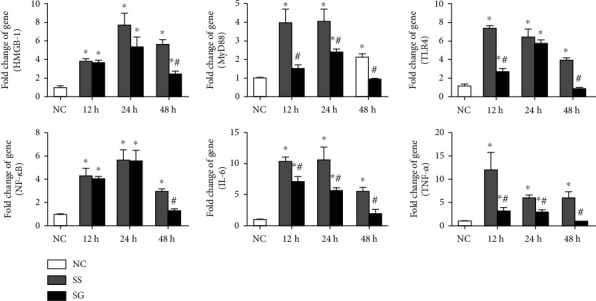
Messenger (m)RNA expressions of high-mobility group box protein-1 pathway-associated genes and subsequent inflammatory cytokines in kidney tissues. NC: normal control; SS: sepsis group with saline; SG: sepsis group with glutamine; HMGB-1: high-mobility group box protein-1; TLR4: toll-like receptor-4; NF-*κ*B: nuclear factor-*κ*B; TNF-*α*: tumor necrosis factor-*α*; IL-6: interleukin-6. mRNA changes were quantitated and analyzed by a real-time PCR and were calculated by the comparative CT (2^-*ΔΔ*Ct^) method. mRNA expression levels in the normal control group were used as a calibrator. Values are expressed as the mean ± SEM. *n* = 8 for each group at 12, 24, and 48 h after cecal ligation and puncture (CLP). Differences between 2 sepsis groups at the same time point were analyzed by *t*-test. The comparison among NC and the sepsis groups at three different time points were analyzed by a one-way analysis of variance (ANOVA) followed by Tukey's post hoc test. ^∗^Significantly differs from the NC group; ^#^significantly differs from the SS group at the same time point (*p* < 0.05).

The HMGB-1, MyD88, TLR4, and NF-*κ*B mRNA expressions were upregulated in the sepsis groups than in the NC group. The gene expression of inflammatory mediators including IL-6 and TNF-*α* was also elevated after sepsis. Compared to the SS group, the SG group had lower gene expressions of MyD88, IL-6, and TNF-*α* at each time point, TLR4 at 12 and 48 h, and HMGB-1 and NF-*κ*B at 48 h after CLP (Figure 2).

### 3.6. mRNA Expressions of Macrophage Infiltration Markers in the Kidney

**Figure 3:**
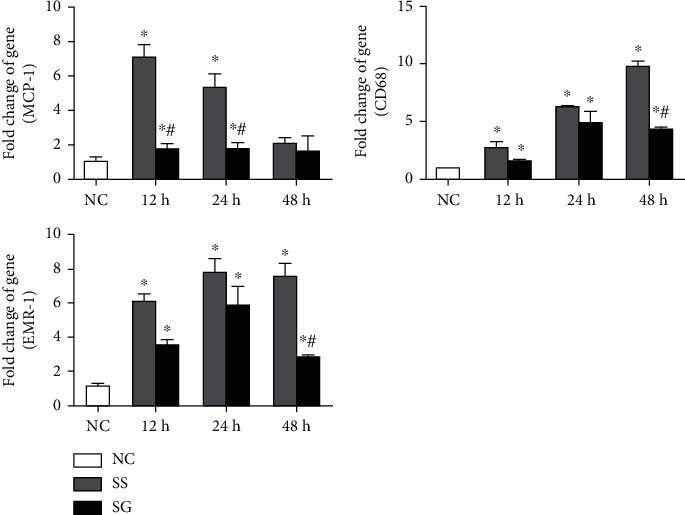
Messenger (m)RNA expressions of macrophage infiltration markers in kidney tissues. NC: normal control; SS: sepsis group with saline; SG: sepsis group with glutamine; CD68: cluster of differentiation 68; EMR-1: epidermal growth factor-like module-containing mucin-like hormone receptor-like-1; MCP-1: monocyte chemoattractant protein-1. mRNA changes were quantitated and analyzed by a real-time PCR and were calculated by the comparative CT (2^-*ΔΔ*Ct^) method. mRNA expression levels in the normal control group were used as a calibrator. Values are expressed as the mean ± SEM. *n* = 8 for each group at 12, 24, and 48 h after cecal ligation and puncture (CLP). Differences between 2 sepsis groups at the same time point were analyzed by *t*-test. The comparison among NC and the sepsis groups at three different time points were analyzed by a one-way analysis of variance (ANOVA) followed by Tukey's post hoc test. ^∗^Significantly differs from the NC group; ^#^significantly differs from the SS group at the same time point (*p* < 0.05).

Compared to the NC, the macrophage infiltration markers, CD68 and EMR-1, and chemoattractant MCP-1 were all elevated after CLP. The MCP-1 expression was significantly lower in the SG group at 12 and 24 h than in the SS group after CLP. The expressions of CD68 and EMR-1 were lower in the SG group than in the SS group at 48 h. No differences in CD68 and EMR-1 were seen at 12 and 24 h post-CLP between the two sepsis groups (Figure 3).

### 3.7. mRNA Expressions of Tight Junction and Injury Proteins in the Kidney

**Figure 4:**
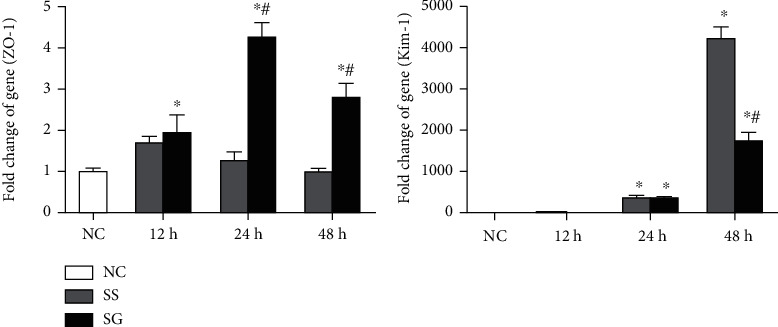
Expressions of genes related to kidney tissue injury. NC: normal control; SS: sepsis group with saline; SG: sepsis group with glutamine; ZO-1: zonula occluden-1; Kim-1: kidney injury molecule. Messenger RNA changes were quantitated and analyzed by a real-time PCR and were calculated by the comparative CT (2^-*ΔΔ*Ct^) method. mRNA expression levels in the normal control group were used as a calibrator. Values are expressed as the mean ± SEM. *n* = 8 for each group at 12, 24, and 48 h after cecal ligation and puncture (CLP). Differences between 2 sepsis groups at the same time point were analyzed by *t*-test. The comparison among NC and the sepsis groups at three different time points were analyzed by a one-way analysis of variance (ANOVA) followed by Tukey's post hoc test. ^∗^Significantly differs from the NC group; ^#^significantly differs from the SS group at the same time point (*p* < 0.05).

The tight junction gene, ZO-1, was significantly elevated in the SG group than in the NC group after sepsis. As compared to the SS group, the SG group exhibited a higher ZO-1 gene expression at 24 and 48 h after CLP. Expression of Kim-1 was increased at 24 and 48 h after CLP. The SG group had a lower Kim-1 expression than the SS group 48 h post-CLP (Figure 4).

### 3.8. Kidney MPO Activities

**Figure 5:**
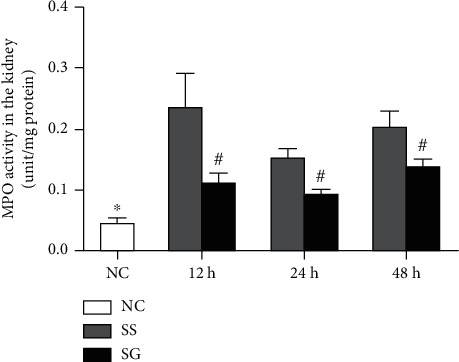
Myeloperoxidase (MPO) activity in kidney tissues. NC: normal control; SS: sepsis group with saline; SG: sepsis group with glutamine. Values are expressed as the mean ± SEM. *n* = 8 for each group at 12, 24, and 48 h after cecal ligation and puncture (CLP). Differences between 2 sepsis groups at the same time point were analyzed by *t*-test. The comparison among NC and the sepsis groups at three different time points were analyzed by a one-way analysis of variance (ANOVA) followed by Tukey's post hoc test. ^∗^Significantly differs from other sepsis groups; ^#^significantly differs from the SS group at the same time point (*p* < 0.05).

MPO activity significantly increased after CLP in both SS and SG groups. Compared to the SS group, the SG group had lower MPO activities at each time point after CLP (Figure 5).

### 3.9. Kidney Lipid Peroxide Concentrations

**Figure 6:**
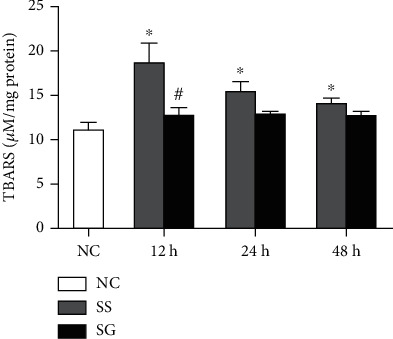
Thiobarbituric acid reactive substance (TBARS) in kidney tissues. NC: normal control; SS: sepsis group with saline; SG: sepsis group with glutamine. Values are expressed as the mean ± SEM. *n* = 8 for each group at 12, 24, and 48 h after cecal ligation and puncture (CLP). Differences between 2 sepsis groups at the same time point were analyzed by *t*-test. The comparison among NC and the sepsis groups at three different time points were analyzed by a one-way analysis of variance (ANOVA) followed by Tukey's post hoc test. ^∗^Significantly differs from NC group; ^#^significantly differs from the SS group at the same time point (*p* < 0.05).

The MDA concentrations were higher in the SS groups than in the NC group, while no differences were noted between the SG and NC groups at each time point after CLP. The SG group had lower MDA levels than the SS group at 12 h post-CLP (Figure 6).

## 4. Discussion

Obesity is a growing challenge around the world. There are increasing numbers of obese patients being admitted to ICUs [24]. There are studies that report that compared to normal weight patients, obesity seems to decrease mortality in the critically ill patients [25, 26]. However, the study also showed that the lower mortality found in the obese patients was abolished when the results were adjusted for comorbidities and sepsis interventions [27]. A clinical study even revealed that mortality of septic patients upon ICU admission was independently associated with obesity [28]. Although discrepancies exist, obesity was found to be correlated with higher infection and exaggerate inflammation [29]. We did not include an obese sham group (without sepsis) in this study, because a former study had shown that compared to the sham group, adipocyte macrophage infiltration and local and systemic inflammation were intensified when obesity coexists with sepsis [30]. Since the kidney is a frequently affected organ in sepsis, the focus of this study was to investigate the impact of GLN on sepsis-induced AKI in obesity. In this study, GLN was administered immediately after CLP. For the group which was sacrificed at 48 h, a second GLN dose was injected 24 h post-CLP. A booster dose was expected to reinforce the efficacy of GLN during the septic state. We found that in the presence of obesity, GLN administration resulted in an elevation in plasma GLN levels and alleviated oxidative stress, and inflammation occurred in the kidney that may protect against sepsis-induced AKI.

We analyzed the gene expressions of HMGB-1-associated mediators. HMGB-1 is a protein released from activated macrophages and damaged tissues which interacts with TLRs [31]. HMGB-1 is considered a mediator of systemic inflammation in the relative late phase of sepsis [32]. Previous studies revealed that endogenous HMGB-1 enhances kidney injury during inflammation [33, 34]. MyD88 is an adaptor protein which acts as a connector between TLRs and the downstream kinases [35]. The HMGB-1-mediated pathway activates cellular signaling that ultimately leads to NF-*κ*B activation and subsequent inflammatory mediator production [31]. Kim-1 is a transmembrane tubular protein expressed on epithelial cells in damaged regions. Kim-1 can be considered as an indicator for AKI [36]. In this study, we observed that the HMGB-1-mediated pathway was upregulated; the inflammatory chemokines and Kim-1 expressions increased in the kidney. Consistent with the elevation of plasma Cre and BUN, NGAL levels increased in the sepsis groups. NGAL is expressed by many tissues including the kidney. Under normal conditions, the expression of NGAL is low, but will significantly increase in epithelial damage and inflammation [37]. Thus, NGAL is treated as a useful biomarker of AKI [38]. The upregulated biomarkers mentioned above indicated that kidney inflammation and injury occur in obesity complicated with sepsis. A study by Kolyva et al. also showed that obesity is associated with enhanced proinflammatory cytokine production and exaggerated systemic oxidative stress in septic patients [39].

Bacterial infection may activate both macrophages and neutrophils. The infiltration of these immune cells into kidney tissue results in persistent inflammation and organ injury [40]. In this study, markers of macrophage were analyzed. CD68 is a transmembrane glycoprotein which is well known as a surface marker abundantly expressed by macrophages in inflamed tissues [41]. The F4/80 molecule, also named EMR-1, was established as a unique marker of murine macrophages [42]. In addition, MPO activities in the kidney were analyzed. MPO is an enzyme released by activated neutrophils. A previous study showed that MPO can be used as a marker of neutrophil infiltration and the severity of inflammation in sepsis patients [43]. In this study, kidney expression of CD68 and EMR-1 was elevated and MPO activity was increased in the obese groups with sepsis, suggesting that macrophage and neutrophil infiltration and inflammation occurred during the experimental period.

In this study, GLN was administered 1 or 2 doses after CLP in obese mice. We observed that there were several favorable effects that were not noted in mice with saline injection. *First*, GLN administration maintained plasma GLN and BCAA levels and reversed sepsis-induced depletion of GLN and glutamate. Previous studies showed that the rates of BCAA oxidation and efflux from muscle tissues are enhanced, thus offering energy substrate for the demand under stress and catabolic conditions [44, 45]. The increased BCAA levels observed in the SS group especially at 12 h after CLP may indicate that more severe catabolism exists at this time point. GLN administration provided more fuel source to fulfill the metabolic needed and alleviated catabolism during sepsis. *Second*, GLN attenuated inflammation at the site of injury and the kidney tissues. We observed that TNF-*α* concentrations reduced while anti-inflammation cytokine IL-10 increased in PLF. Also, the HMGB-1-mediated pathway was downregulated and inflammatory cytokine expressions reduced in the kidneys. Our results are consistent with previous studies that GLN downregulated the HMGB-1 pathway and inhibited NF-*κ*B activation and subsequent downstream target gene expressions [19, 46]. In addition, GLN can activate the expression of the peroxisome proliferator-activated receptor (PPAR). PPAR-*γ* can be activated by several ligands and has an anti-inflammatory property. GLN is one of the ligands of PPAR-*γ*. A previous study found that GLN administration to the intestinal lumen induced the expression of PPAR-*γ* and protected against intestinal inflammation in the postischemic gut [47]. *Third*, GLN administration reduced macrophage and neutrophil infiltration in the kidney. Markers of activated macrophage and neutrophil, including CD68, EMR-1 expression, and MPO activity in kidneys, decreased in the SG group. Consistent with the findings are lower plasma NGAL levels and kidney Kim-1 and MCP-1 whereas higher ZO-1 expressions were noted suggesting that sepsis-associated kidney injury was attenuated when GLN was administered. Previous studies found that GLN sustained the T cell population and modulated a more balanced T helper cell polarization in sepsis that is associated with attenuating inflammation and kidney injury [16, 18].

Despite the anti-inflammatory property of GLN, there may have been other mechanisms that participated in attenuating kidney injury in this study. Oxidative stress and inflammation are underlying disorders occur in both obesity and sepsis [48, 49]. Obesity concurrence with sepsis further intensifies the inflammatory reaction and oxidative stress [29]. GLN is the precursor of an endogenous antioxidant, glutathione [50]. In this study, we found that the production of kidney lipid peroxides was reduced in the SG group. The lower oxidative stress exerted from GLN-associated redox reactions may play a role in alleviating kidney injury. On the other hand, vascular endothelium damage and dysfunction are correlated with the progression of multiorgan injury in sepsis [51]. Endothelial progenitor cells (EPCs) derived from bone marrow are capable of proliferating and differentiating into mature endothelial cells [52]. Circulating EPCs were shown to be involved in repairing and maintaining vascular endothelium integrity during sepsis [53]. A previous study showed that GLN administration after CLP promotes EPC mobilization, improves vascular function, and protects remote organ injury against sepsis [54]. However, whether GLN administration may initiate endogenous endothelium repair and improve microvascular perfusion, thus alleviating kidney injury, requires further investigation.

**Figure 7:**
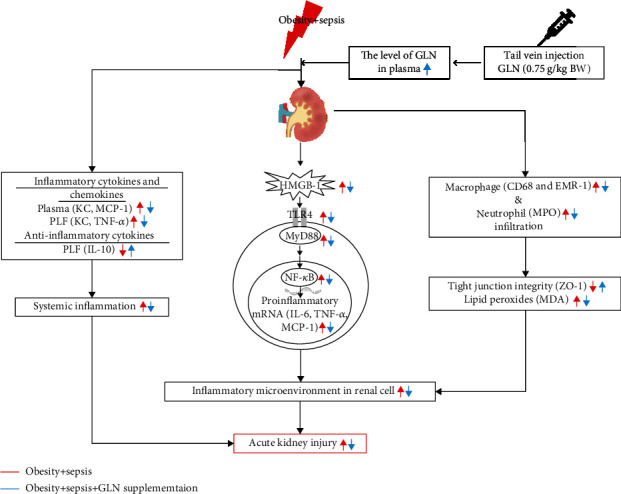
The proposed mechanisms of glutamine (GLN) regulation on attenuating sepsis-induced acute kidney injury (AKI). Obesity exaggerates the severity of sepsis, and AKI is a common complication. Underlying sepsis, immune cells release inflammatory cytokines and chemokines that result in systemic inflammation. In the kidney, upregulation of HMGB-1 activates the NF-*κ*B pathway and subsequent downstream inflammatory mediator production. Activated macrophage and neutrophil infiltration into kidney tissue may worsen the integrity of tight junction and increase the production of lipid peroxides. The inflammatory microenvironment within renal cells ultimately leads to AKI. GLN administration increases the plasma levels of GLN, blocks the inflammatory pathways, and reduces lipid peroxide production, thus ameliorating the occurrence of AKI. Red line means the effects of obesity complicated with sepsis. Blue line means the effects of GLN administration on obesity complicated with sepsis. CD68: cluster of differentiation 68; CLP: cecal ligation and puncture; EMR-1: epidermal growth factor-like module-containing mucin-like hormone receptor-like-1; HMGB-1: high-mobility group box protein-1; KC: keratinocyte-derived chemokine; IL: interleukin; MCP-1: monocyte chemoattractant protein-1; MDA: malondialdehyde; MPO: myeloperoxidase; MyD88: myeloid differentiation factor 88; NF-*κ*B: nuclear factor-*κ*B; PLF: peritoneal lavage fluid; TLR4: toll-like receptor-4; TNF-*α*: tumor necrosis factor-*α*; ZO-1, zonula occluden-1.

As far as we know, this study investigated the effects of GLN on sepsis-induced kidney inflammation and injury in obesity for the first time. Results showed that coexisting with obesity, sepsis resulted in macrophage and neutrophil infiltration in the kidney that may lead to inflammation and injury of the organ. GLN administration after CLP increased plasma GLN levels, attenuated immune cell infiltration in the kidney, and resolved sepsis-induced inflammation and AKI. The proposed mechanisms of GLN regulation on attenuating sepsis-induced AKI is presented as Figure 7. The results presented here showed that GLN may have potential to attenuate inflammation and protect against kidney injury in obesity complicated with sepsis. This model may have clinical application for obese patients with a risk for infection in abdominal surgery.

## Figures and Tables

**Table 1:** Composition of the high-fat diet.

Ingredient	g/kg
Casein	259.13
L-Cysteine	3.89
Maltodextrin	161.96
Sucrose	89.14
Cellulose	64.78
Soybean oil	32.39
Lard	317.44
Mineral mix^1^	12.96
Dicalcium phosphate	16.84
Calcium carbonate, 1H_2_O	7.13
Potassium citrate	21.38
Vitamin mix^2^	12.96
Total	1000

^1^The composition of the mineral mixture is listed as follows (mg/g): calcium phosphate dibasic, 500; sodium chloride, 74; potassium sulfate, 52; magnesium oxide, 24; potassium citrate monohydrate, 20; manganese carbonate, 3.5; ferric citrate, 6; chromium potassium sulfate, 0.55; zinc carbonate, 1.6; cupric carbonate, 0.3; potassium iodate, 0.01; and sodium selenite, 0.01. ^2^The composition of the vitamin mixture is listed as follows (mg/g): DL-*α*-tocopherol acetate, 20; nicotinic acid, 3; retinyl palmitate, 1.6; calcium pantothenate, 1.6; pyridoxine hydrochloride, 0.7; thiamin hydrochloride, 0.6; riboflavin, 0.6; cholecalciferol, 0.25; D-biotin, 0.05; menaquinone, 0.005; and cyanocobalamin, 0.001.

**Table 2:** Sequences of oligonucleotide primers used for PCR amplification.

Gene name	Primer sequence (5′→3′)	Accession no.
NF-*κ*B	F: TTAGCCAGCGAATCCAGACC	M61909.1
	R: AGTTCCGGTTTACTCGGCAG	
HMGB-1	F: CCATTGGTGATGTTGCAAAG	NM_010439.4
	R: CTTTTTCGCTGCATCAGGTT	
MyD88	F: CATGGTGGTGGTTGTTTCTGAC	NM_010851.2
	R: TGGAGACAGGCTGAGTGCAA	
TLR4	F: AGAAATTCCTGCAGTGGGTCA	NM_021297.2
	R: TCTCTACAGGTGTTGCACATGTCA	
Kim-1	F: GCATCTCTAAGCGTGGTTGC	NM_134248.2
	R: TCAGCTCGGGAATGCACAA	
MCP-1	F: GATTCACATTTGCGCTGCCT	U12470.1
	R: TGAGCCTGGGAGATCACCAT	
TNF-*α*	F: ATGGCCTCCCTCTCATCAGT	NM_013693.3
	R: TTTGCTACGACGTGGGCTAC	
IL-6	F: TCCTACCCCAACTTCCAATGCTC	NM_012589.1
	R: TTGGATGGTCTTGGTCCTTAGCC	
CD68	F: TGTTCAGCTCCAAGCCCAAA	NM_001291058.1
	R: ACTCGGGCTCTGATGTAGGT	
EMR-1	F: ACCTTGTGGTCCTAACTCAGTC	U66889.1
	R: ACAAAGCCTGGTTGACAGGTA	
ZO-1	F: GATGTTTATGCGGACGGTGG	BC138028.1
	R: AAATCCAAACCCAGGAGCCC	
GAPDH	F: AACGACCCCTTCATTGAC	M32599.1
	R: TCCACGACATACTCAGCAC	

NF-*κ*B: nuclear factor-*κ*B; HMGB-1: high-mobility group box protein-1; MyD88: myeloid differentiation factor 88; TLR4: toll-like receptor-4; Kim-1: kidney injury molecule-1; MCP-1: monocyte chemoattractant protein-1; TNF-*α*: tumor necrosis factor-*α*; IL-6: interleukin-6; CD68: cluster of differentiation 68; EMR-1: epidermal growth factor-like module-containing mucin-like hormone receptor-like-1; ZO-1, zonula occluden-1; GAPDH: glyceraldehyde 3-phosphate dehydrogenase.

**Table 3:** Plasma concentrations of kidney function marker and inflammatory chemokine among groups.

	NC	SS12h	SG12h	SS24h	SG24h	SS48h	SG48h
NGAL (*μ*g/dL)	0.08 ± 0.01^∗^	3.52 ± 1.84	2.76 ± 1.08	52.3 ± 5.40	35.1 ± 6.50^#^	39.5 ± 32.70	48.0 ± 31.60
BUN (mg/dL)	18.9 ± 0.90^∗^	67.4 ± 8.40	69.1 ± 6.30	97.1 ± 6.10	102.3 ± 6.10	139.9 ± 14.40	130.0 ± 29.30
Cre (mg/dL)	0.09 ± 0.01^∗^	0.14 ± 0.03	0.12 ± 0.01	0.70 ± 0.09	0.71 ± 0.10	1.10 ± 0.28	1.20 ± 0.32
KC (ng/mL)	0.21 ± 0.04^∗^	137.3 ± 36.4	27.9 ± 6.6^#^	226.4 ± 143.3	190.3 ± 97.1	4.57 ± 2.15	6.49 ± 1.08
MCP-1 (ng/mL)	0.03 ± 0.004^∗^	2.68 ± 1.96	2.08 ± 1.23	2.70 ± 1.50	3.70 ± 0.18	3.93 ± 3.88	3.14 ± 2.57

Data are presented as the mean ± SEM. NC: normal control group; SS: sepsis group with saline injection sacrificed at 12, 24, and 48 h after cecal ligation and puncture (CLP); SG: sepsis group with glutamine injection sacrificed at 12, 24, and 48 h after CLP; NGAL: neutrophil gelatinase-associated lipocalin-2; BUN: blood urea nitrogen; Cre: creatinine; KC: keratinocyte-derived chemokine; MCP-1: monocyte chemoattractant protein. Differences between 2 sepsis groups at the same time point were analyzed by *t*-test. The comparison among NC and the sepsis groups at three different time points were analyzed by a one-way analysis of variance (ANOVA) followed by Tukey's post hoc test. ^∗^Significantly differs from other sepsis groups; ^#^significantly differs from the SS group at the same time point (*p* < 0.05).

**Table 4:** The concentrations of inflammatory cytokines and chemokines in peritoneal lavage fluid.

	NC	SS12h	SG12h	SS24h	SG24h	SS48h	SG48h
TNF-*α* (pg/mg protein)	N.D.	4.88 ± 2.10	4.36 ± 0.84	26.1 ± 2.8	20.8 ± 5.3	46.9 ± 13.2	18.7 ± 5.1^#^
IL-10 (pg/mg protein)	N.D.	47.8 ± 36.1	46.0 ± 29.7	309.5 ± 97.7	693.0 ± 85.1^#^	89.0 ± 66.9	95.2 ± 69.1
KC (ng/mg protein)	0.01 ± 0.003^∗^	9.67 ± 0.69	3.39 ± 1.36^#^	6.91 ± 0.63	5.36 ± 0.44	2.70 ± 1.36	1.19 ± 0.45
MCP-1 (ng/mg protein)	0.08 ± 0.001^∗^	5.17 ± 0.86	2.45 ± 0.74^#^	2.73 ± 0.63	2.46 ± 0.82	2.56 ± 1.12	2.03 ± 0.90

Data are presented as the mean ± SEM. The grouping of the experiment is described in the footnote of Table 1. N.D.: nondetectable; IL-10: interleukin-10; TNF-*α*: tumor necrosis factor-*α*; KC: keratinocyte-derived chemokine; MCP-1: monocyte chemoattractant protein. Differences between 2 sepsis groups at the same time point were analyzed by *t*-test. The comparison among NC and the sepsis groups at three different time points was analyzed by a one-way analysis of variance (ANOVA) followed by Tukey's post hoc test. ^∗^Significantly differs from other sepsis groups; ^#^significantly differs from the SS group at the same time point (*p* < 0.05).

## Data Availability

The data used to support the findings of this study are available from the corresponding author upon request.
